# Tracheal Injury Following Video-Assisted Thoracoscopy Surgery Lobectomy

**DOI:** 10.7759/cureus.100709

**Published:** 2026-01-03

**Authors:** Bothayna Amien, Habiba Othman, Syed S Qadri

**Affiliations:** 1 Cardiothoracic Surgery, Hull University Teaching Hospitals NHS Trust, Hull, GBR; 2 College of Medicine, Alfaisal University, Riyadh, SAU

**Keywords:** emphysema, endotracheal intubation complications, management of tracheal injury, tracheal injury, endotracheal intubation

## Abstract

This case report highlights a rare complication encountered in thoracic surgery following endotracheal (ET) intubation. The patient underwent lobectomy for a right lower lobe lung lesion, during which a tracheal injury above the level of the carina was identified at the end of the procedure. It was immediately managed with surgical repair. This case emphasises the importance of early detection and prompt management. The patient survived and made a good recovery; we describe the diagnostic process and management undertaken.

## Introduction

Tracheal injuries may be iatrogenic or traumatic; although rare, they can be life-threatening [1]. The incidence of tracheal injury among patients admitted to major trauma centres with chest trauma in the UK is approximately 0.18%, with these injuries contributing to prolonged hospital stay [2]. The true incidence of iatrogenic tracheal injury is difficult to determine; however, a rate of 0.15% has been reported in single-centre studies following tracheal intubation with double-lumen tubes [3]. An incidence of 0.000013% has been reported for single-lumen tube intubations in a multicentre study conducted across hospitals in Germany [1]. The overall mortality associated with tracheal injuries following intubation has been reported as 22% [4].

The most commonly injured part is the posterior membranous portion of the trachea due to the absence of cartilaginous support. Several factors may contribute to this injury, including endotracheal (ET) tube size, age, sex, and body mass index (BMI) [5]. In this report, we discuss this complication, including its presentation and management, in the context of thoracic surgery in a patient who underwent lobectomy.

## Case presentation

**Figure 1:**
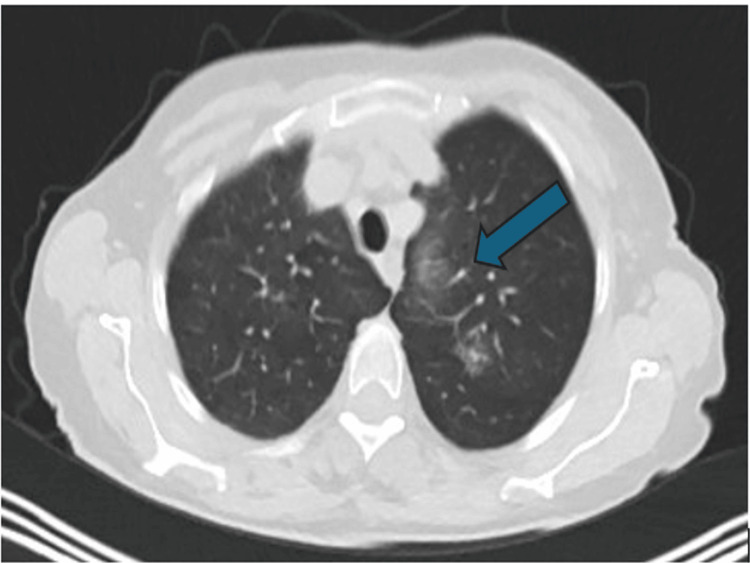
CT scan showing right lower lobe lesion

A 69-year-old lady was referred to thoracic surgery for a slowly growing, positron emission tomography (PET)-avid lesion in the right lower lobe (Figure 1). The patient had only symptoms of intermittent cough, no loss of weight or appetite. Radiological diagnosis T1b N0 M0 was made. She was a current smoker with borderline lung function: forced expiratory function in one second (FEV1), 2.05 (51% predicted); forced vital capacity (FVC), 2.5 (95% predicted); transfer factor for carbon monoxide (TLCO), 78% predicted; carbon monoxide transfer coefficient (KCO), 83% predicted. She had a past medical history of chronic obstructive pulmonary disease (COPD) and carpal tunnel syndrome. The patient was independent with activities of daily living (ADL), with a BMI of 21.1 kg/m^2^. She was offered curative surgical resection of the tumour.

**Figure 2:**
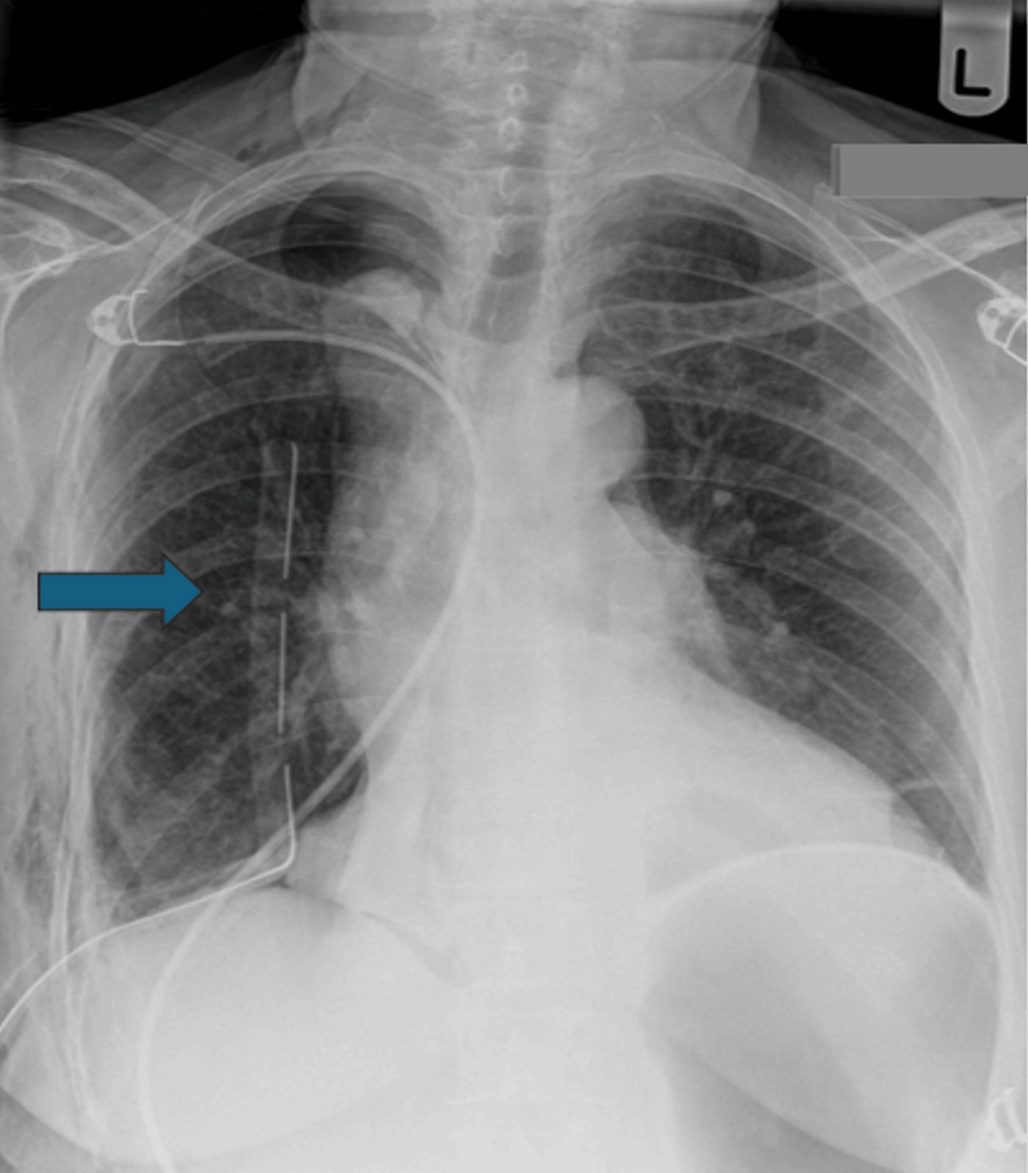
CXR showing postoperative expanded lung and drain in correct place CXR: chest radiograph

Patient underwent right video-assisted thoracoscopy surgery (VATS) lower lobe resection with a 35 French unit (Fr) double-lumen ET tube. Three portal VATS performed, fragile tissues were noted along with several bullae. Standard lobectomy performed; however, surgical emphysema was noted inside tissues around the oesophagus and pericardium at the end of the surgery. Nasogastric (NG) tube inserted to aspirate for air, but nothing is present. Washout was done, which identified an air leak above the azygous vein area. Decision made to convert to thoracotomy to explore further, a 5-6 cm tear identified in the trachea posteriorly from the edge of tracheal rings to the posterior wall, 1 cm above the carina. Team decision made to change the double lumen to a single lumen in the left bronchus, which would allow us to ventilate the left side while working on the right-side repair and avoid the damage that could be further caused by the ET tube on the right side. Therefore, the patient was put in a supine position, and the tube was changed. There was an episode of decreasing oxygen saturations, which raised suspicions for tension pneumothorax on the left side; however, needle decompression was negative. The tube was placed, and the saturations were back to normal. The patient was positioned in the left lateral decubitus position again, and the tear was repaired under direct vision using 4-0 Prolene suture. An intercostal muscle flap was used to reinforce the repair. Washout was done again, and no air leak was identified. A 28 Fr drain was inserted, and the thoracotomy was closed. Post-procedure chest radiograph (CXR) showed good lung expansion and a drain in place (Figure 2). The patient was monitored on the intensive therapy unit (ITU) following the procedure and was placed on high-flow nasal oxygen. Patient recovered well with physiotherapy; however, she started to complain of difficulty swallowing and was seen by the speech and language therapy (SALT) team. Video fluoroscopy showed no evidence of strictures or oesophageal damage. The patient gradually improved and was discharged in a week after her procedure.

## Discussion

Tracheal injuries are a rare complication that can occur following ET intubation [4]. Early identification and management are important for good recovery. Schneider et al. reported a multicentre study across hospitals in Germany, which showed that 604 of 1,033 tracheal injuries were iatrogenic. The most common aetiology was tracheal intubation and mechanical ventilation, accounting for 61.4% of cases [1].

One of the earliest case series was reported by Borasio et al., who described 10 cases identified over a seven-year period beginning in 1997. The majority of patients were female (nine women and one man), with a mean age of 50 years. Although the intubations were described as uneventful, tracheal rupture was associated with the use of double-lumen tubes, and surgical emphysema was the most common presentation [6].

The most frequently reported risk factors in the literature include female sex, age over 50 years, short neck, use of double-lumen tubes, high BMI, and repeated intubation attempts. Common clinical presentations of tracheal injury include subcutaneous emphysema, dyspnoea, pneumothorax, and pneumomediastinum, with subcutaneous emphysema being the earliest and most frequent sign [1,3,4].

A systematic review by Miñambres et al. demonstrated that 86.2% of patients with tracheal injury following ET intubation were females, with a mean age of 50 years. In this review, 17% of injuries were diagnosed intraoperatively, while 82.4% had a delayed diagnosis. Subcutaneous emphysema was the most common symptom, followed by pneumomediastinum, dyspnoea, and pneumothorax. The overall reported mortality was 22% [4].

Management of tracheal injuries may be surgical or conservative, depending on the timing of diagnosis and the patient’s clinical condition. Schneider et al. reported that the rate of surgical repair for intubation-related tracheal injuries was 67% in institutions providing maximum care and in university hospitals [1].

In our case, the patient sustained a tracheal injury following intubation with a double-lumen tube for lobectomy surgery. The injury was identified intraoperatively when tissue emphysema became evident. In such scenarios, withdrawal of the tube to facilitate suturing of the tear should be considered. A multidisciplinary team decision was made to reposition the patient from the left lateral decubitus position to the supine position to allow safe placement of a single-lumen ET tube to ventilate the left side. During this process, close monitoring of haemodynamic stability is essential, and the team should be ready for management of contralateral pneumothorax if encountered. Conversion to thoracotomy is often necessary to improve visualisation and accurately identify the tracheal tear. The choice of needle is important due to the limited operative space; repair with 4-0 Prolene sutures is recommended. Care must be taken during suturing to avoid oesophageal injury, and surgeons should remain vigilant for this potential complication. In this case, an intercostal muscle flap was used to reinforce the repair.

We believe this injury resulted from a combination of a narrow trachea and fragile tissues, as a result of ET tube placement and cuff inflation at the start of the procedure. This highlights the importance of careful selection of ET tube size, particularly in smaller patients. Prompt recognition and careful planning allowed the complication to be managed safely, resulting in a good outcome.

## Conclusions

This case report demonstrates the importance of immediate management of tracheal injury in the context of thoracic surgery. It also emphasises the role of prompt and effective decision-making, as well as multidisciplinary team involvement. Based on our experience and the favourable patient outcome, we recommend early and timely management of intraoperative tracheal injury.
